# Evolving and Novel Applications of Artificial Intelligence in Cancer Imaging

**DOI:** 10.3390/cancers17091510

**Published:** 2025-04-30

**Authors:** Mustaqueem Pallumeera, Jonathan C. Giang, Ramanpreet Singh, Nooruddin S. Pracha, Mina S. Makary

**Affiliations:** 1 The Ohio State University College of Medicine, Columbus, OH 43210, USA; mustaqueem.pallumeera@osumc.edu (M.P.); nooruddin.pracha@osumc.edu (N.S.P.); 2 Northeast Ohio Medical University, Rootstown, OH 44272, USA; jgiang@neomed.edu (J.C.G.); rsingh10@neomed.edu (R.S.); 3 Division of Vascular and Interventional Radiology, Department of Radiology, The Ohio State University Wexner Medical Center, Columbus, OH 43210, USA

**Keywords:** radiology, artificial intelligence, cancer, imaging

## Abstract

Artificial intelligence (AI) is revolutionizing cancer imaging, enhancing screening, diagnosis, and treatment options for clinicians. AI-driven applications, particularly deep learning and machine learning, excel in risk assessment, tumor detection, classification, and predictive treatment prognosis. Machine learning algorithms, especially deep learning frameworks, improve lesion characterization and automated segmentation, leading to enhanced radiomic feature extraction and delineation. Radiomics, which quantifies imaging features, offers personalized treatment response predictions across various imaging modalities. AI models also facilitate technological improvements in non-diagnostic tasks, such as image optimization and automated medical reporting. Despite advancements, challenges persist in integrating AI into healthcare, tracking accurate data, and ensuring patient privacy. Validation through clinician input and multi-institutional studies is essential for patient safety and model generalizability. This requires support from radiologists worldwide and consideration of complex regulatory processes. Future directions include elaborating on existing optimizations, integrating advanced AI techniques, improving patient-centric medicine, and expanding healthcare accessibility. AI can enhance cancer imaging, optimizing precision medicine and improving patient outcomes. Ongoing multidisciplinary collaboration between radiologists, oncologists, software developers, and regulatory bodies is crucial for AI’s growing role in clinical oncology. This review aims to provide an overview of the applications of AI in oncologic imaging while also discussing their limitations.

## Simple Summary

Artificial intelligence (AI) is revolutionizing cancer imaging, enhancing screening, diagnosis, and treatment options. AI-driven applications assess risk, detect tumors, classify them, and predict treatment outcomes. Machine learning algorithms improve lesion characterization and automated segmentation to enhance radiomic feature extraction, quantifying imaging features for personalized treatment response predictions. AI models facilitate technological improvements in image optimization, complex data integration, and automated medical reporting. Despite advancements, challenges persist in integrating AI into healthcare, tracking accurate data, and ensuring patient privacy. Rigorous validation through clinician input and multi-institutional studies is essential for patient safety and model generalizability. Future directions include elaborating on optimizations, integrating advanced AI techniques, improving patient-centric medicine, and expanding healthcare accessibility. AI significantly enhances cancer imaging, optimizing precision medicine and improving patient outcomes. Ongoing multidisciplinary collaboration is crucial for AI’s growing role in clinical oncology. This article reviews AI-driven applications in cancer imaging, current limitations, and future directions.

## 1. Introduction

Diagnostic radiology has evolved greatly over the years, with advances leading to increases in diagnostic accuracy and radiologists having greater involvement in clinical decision-making [1]. This has led to an ever-growing utilization of medical imaging, with radiology playing an increasingly vital role in healthcare [2]. As the demand for imaging continues to rise, the limited supply of radiologists is facing a rapidly increasing work burden. Projections estimate imaging utilization by 2055 to be 16.9 to 26.9% higher than 2023 levels, with continuation of recent per-person utilization trending towards broadening this range [3]. The development of artificial intelligence (AI) has offered a promising solution to many of the challenges faced by radiologists. AI is generally recognized as a technology that can imitate human cognitive functions, and machine learning (ML) is a subclass of AI which allows it to improve with experience. Deep learning (DL) is a further subclass of ML which allows algorithms to train themselves [4].

**Figure 1:**
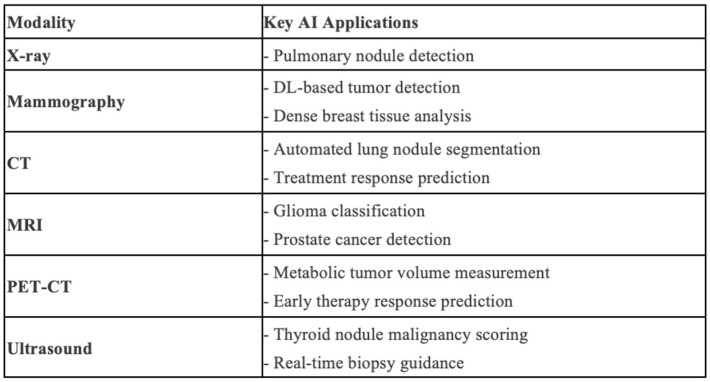
Key applications of AI for each imaging modality.

Cancer imaging in particular is an area in which early exploration of these tools can improve work efficiency, reduce errors, and enhance diagnostic performance [5]. These technologies can be integrated into many imaging modalities, including X-rays, computed tomography (CT), positron emission tomography (PET), magnetic resonance imaging (MRI), and mammography (Figure 1). For example, in chest radiography, AI implementation into chest radiograph interpretation shows promising accuracy in detecting pulmonary nodules, with the development of more accurate algorithms aiming to match radiologist readings [6]. In abdominal imaging, integration of the models into CT scan analysis assists in reducing segmentation errors and eliminating peripheral structures from consideration with a Dice coefficient of 88.55%, outperforming state-of-the-art techniques [7]. The Dice coefficient is used in AI for medical image segmentation as a quantifiable metric to measure how well an algorithm’s output matches radiologists’ annotations [8]. Integration into PET scan analysis can help reduce acquisition time and improve image quality with denoising and deblurring techniques [9]. In neuroradiology, AI integration into MRI interpretation can help differentiate low-grade gliomas from high-grade with an area under the curve (AUC) of 93.2% [10]. AI in mammography has significantly enhanced the accuracy and effectiveness of breast cancer detection with an AUC of 89.6% [11]. These applications are continuously being refined and implemented ever deeper into each modality in order to increase clinical efficacy.

**Figure 2:**
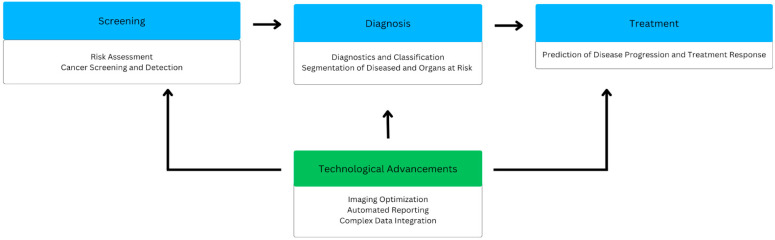
Summary of the AI applications in cancer imaging in screening, diagnosis, and treatment.

However, with many technological advancements being developed independently of one another, it can be a challenge to achieve routine clinical application. As these tools are undergoing continued development, it is important to assess the clinical areas in which they can facilitate screening, diagnosis, and treatment within oncology radiology. This review aims to provide an overview of the applications of AI in oncologic imaging and how it is reshaping the clinical practice of cancer treatment, highlighting the advances made in clinical efficacy through the discussion of foundational and up-to-date literature while also discussing their limitations (Figure 2). Limitations of AI as a whole, as well as future directions for the technology, will also be explored.

## 2. Screening

### 2.1. Risk Assessment

Within preventative healthcare, it is important to assess individuals for their risk of developing health conditions. Risk-based cancer screening is defined as screening eligibility based on the personal risk of individuals [12]. AI tools can be utilized in imaging to aid in better assessing patients’ risk of developing a malignancy in the future.

Breast density assessment is an important tool used to assess the relative risk of developing breast cancer. Due to the qualitative nature of the assessment, there is variability in density categorization ranging between 6.3% and 84.5%, with disagreements in classification between “not dense” and “dense” being as high as 17.2% [13]. A DL system demonstrated high breast density classification accuracy with an AUC of 0.9882 for difficult-to-distinguish breast densities, significantly enhancing the clinical assessment of breast cancer screening. However, further testing must be carried out as the study only included cancer-free images, as cancer-affected scans could introduce bias into the model [14]. The incorporation of AI in breast imaging could help identify women at risk for missed breast cancer during screening and identify those who would benefit the most from additional imaging [15]. Recent incorporations of AI into risk assessment found it to effectively identify women with higher breast cancer risk in an intermediate-risk population, including women with mammographically occult cancers and women with prior breast cancer, with AUCs of 0.72 and 0.81, respectively. However, during testing, there was a probability of false-negative results, underscoring the importance of continued refinement of these models [16]. AI systems like Transpara can identify high-risk mammographic features years before clinical diagnosis, with one study finding that 38% of screen-detected cancers were flagged as high-risk on prior imaging [17]. However, this study was retrospective and may not reflect real-world performance, as it relied on preselected cases rather than prospective screening populations. Workflow optimization has also been demonstrated, with a multicenter trial reporting that AI reduced the radiologist workload by 56.7% through low-risk case triaging [18]. However, this trial’s generalizability may be limited, as it was conducted in a single healthcare system with standardized imaging protocols.

For lung cancer, AI has improved nodule detection sensitivity (56.4–95.7%) and, when combined with LUNG-RADS, achieved an AUC of 0.89 for malignancy prediction [19]. However, the wide sensitivity range reflects the variability across institutions, and the study noted poorer performance for nodules in challenging anatomical locations.

Development of cancer predictive models is extending to urologic malignancies as well. Meanwhile, AI models show a promising future in predicting muscle-invasive bladder cancer, with an area under the curve (AUC) of 0.85 (95% CI, 0.81–0.88) for CT, 0.92 (95% CI, 0.89–0.94) for MRI, 0.89 (95% CI, 0.86–0.92) for radiomics, and 0.91 (95% CI, 0.88–0.93) for DL. However, the results showed that the studies had poor overall quality, with a median Checklist for Artificial Intelligence in Medical Imaging (CLAIM) study adherence rate of 64.1%, a median radiomics quality score percentage of 30.6%, and a high risk of bias among all studies [20]. For prostate cancers, DL can be incorporated as an independent diagnostic marker for risk calculators in order to improve patient selection prior to biopsy with clinical and demographic information. AI incorporation into a risk calculator spared 49% of biopsies while maintaining a high negative predictive value of 94%, compared to the prostate imaging reporting data system which spared 37% of biopsies. The model was trained on biopsy-naive patients, however, meaning the use of DL in active surveillance to predict tumor progression risk remains to be investigated [21]. Without a doubt, as the development of AI models progresses, deeper integration into cancer risk assessments will follow.

### 2.2. Cancer Detection

Cancer screening is another area where AI algorithms can improve sensitivity and specificity by analyzing large datasets of imaging and biomarker data to improve clinical efficacy [22]. For breast cancer screening, it has been demonstrated that AI algorithms can match the performance of expert readers and even surpass them with an absolute margin of 11.5%. Models can also assist second readers for screening mammographic reviews by reducing their workload by 88% [23]. AI can also assist in triaging for prioritizing image reading, which is a technology that women undergoing mammographic screening find acceptable [24,25]. In the context of pulmonary cancer, AI models can analyze nodules on CT imaging to identify and predict EGFR mutational status non-invasively and aid in triaging patients for targeted therapies. An average precision at 50% intersection over union of 70.19% was achieved in detecting the location and properties of nodules within the lung region of interest, demonstrating high efficacy. However, due to the lack of external annotated data and the datasets being sourced from solely one institution, further development must be carried out to improve generalizability [26]. With metastatic prostate carcinoma, a study by Shao et al. found high values for mean training accuracy (99.51%), mean testing accuracy (81.70%), mean testing sensitivity (80.63%), and mean testing specificity (82.82%) in screening for metastasis [27]. Giganti et al. validated a separate model across six institutions with diverse hardware, achieving an AUC of 0.91, sensitivity of 95%, and specificity of 67%, confirming the generalizability and reproducibility of high-performing AI in routine prostate imaging. These tools reduce inter-reader variability and can support biopsy triage in settings lacking subspecialty expertise. Limitations include the use of biopsy rather than whole-mount pathology as ground truth, incomplete biopsy verification for MRI-negative cases, reliance on radiologist-identified lesions, potential selection bias due to population homogeneity, and limited scanner diversity, all of which may affect the model’s sensitivity, specificity, and generalizability [28].

While AI assistance may improve the sensitivity of cancer screening, it also increases the number of false positives and unnecessary surveillance for patients in its current state [29]. Further development and research will aid in not only expanding the types of cancers that can be screened for, but also the efficacy at which they are screened.

## 3. Diagnosis

### 3.1. Diagnostics and Classification

ML systems have the potential to go further than disease detection, utilizing processes to enhance the classification of imaging findings in cancer. In a study performed by Ma et al., it was illustrated that ML algorithms were able to accurately distinguish triple-negative breast cancer (TNBC) from other molecular subtypes, such as ER/PR-positive tumors and HER2-positive tumors. A decision tree model examining mammography and ultrasound images showed the best performance with an AUC of 0.971 and accuracy of 0.947. However, the study was limited by its retrospective nature and by being conducted in a single institution, lending itself to selection bias. Only five ML algorithms were discussed, and the study focused on the dichotomy of the molecular subtype of breast cancer, not exploring the four classifications of the molecular subtype of breast cancer [30]. Chen et al. addressed inter-reader variability in breast density classification using a deep learning model trained on over 57,000 mammograms, achieving over 90% accuracy, a Kappa of 0.803, and inference times 500× faster than those of radiologists. Limitations include the underrepresentation of certain breast density categories due to screening data from primary hospitals, the absence of a clinical context during radiologist evaluations, and the use of subjectively defined BI-RADS density categories as ground truth rather than objective imaging measures [31]. Despite these limitations, these studies showed the potential of using AI algorithms to determine molecular subtypes of breast cancer using mammography and ultrasound findings instead of relying solely on invasive immunohistochemical detection methods.

Stanford’s MUSK (Multimodal transformer with Unified maSKed modeling) model further enhances radiology-based cancer classification by fusing imaging features with clinical and molecular data. For tumor subtyping, it distinguishes lung adenocarcinoma (textural heterogeneity, AUC 0.80) from squamous cell carcinoma on CT and predicts EGFR mutations (75% accuracy) via ground-glass opacity patterns. In brain MRI, it classifies IDH-mutant gliomas (85% accuracy) using T2-FLAIR signals (1). For differential diagnosis, MUSK differentiates clear-cell RCC from angiomyolipomas on MRI (89% accuracy) and osteosarcoma from Ewing sarcoma on X-rays. While promising, clinical adoption requires validation in prospective trials and mitigation of biases from underrepresented populations [32].

In a study by Xie et al., a deep learning system (UC-STTNet) utilizing dual-modal ultrasound (US) and clinical data achieved an area under the curve (AUC) of 0.89 for diagnosing malignant soft tissue tumors (STTs), outperforming junior and intermediate radiologists while matching senior radiologists’ accuracy [33]. This AI tool not only improved diagnostic consistency but also reduced operator dependence, highlighting its utility in clinical settings. Similarly, Liu et al. developed a PET/CT radiomics model using LightGBM to differentiate cervical adenocarcinoma (AC) from squamous cell carcinoma (SCC), achieving an AUC of 0.851 in internal validation [34]. The superior performance of PET-derived radiomics over CT features underscores AI’s ability to leverage metabolic imaging for precise tumor subtyping, which could guide personalized treatment strategies. In another study, Hu et al. applied CT texture analysis to classify testicular masses, with logistic regression models yielding AUCs of 0.946 (benign vs. malignant) and 0.982 (lymphoma vs. non-lymphoma) [35]. These findings illustrate AI’s role in non-invasive preoperative assessment, aiding in clinical decision-making for surgery or chemotherapy.

Collectively, these studies underscore AI’s transformative impact on oncology diagnostics, offering high-accuracy, non-invasive alternatives to traditional biopsies while addressing challenges such as inter-observer variability and diagnostic delays.

### 3.2. Segmentation of Disease and Organs at Risk

Segmentation, or the outlining of disease, is vital to the classification of radiological images and essential for many AI/ML and radiomics studies. Extracting quantitative tumor measurements, delineating tumor borders for radiotherapy planning, and tracking segmentation across time can all inform clinicians on the size of a tumor and the effect of treatment on tumor burden. A study by Dinkel et al. found that manual tracing of lesion borders resulted in greater inter-reader variability compared to that in AI models utilizing automatic disease segmentation. The variation coefficient for manual measurement compared to semi-automated measurement was 15.7% vs. 7.3% using the World Health Organization guidelines and 7.7% vs. 2.7% using the Revised Response Evaluation Criteria in Solid Tumors guidelines [36].

Disease segmentation algorithms are relatively well developed for specific diseases and image types, likely due to the efficiency of ML when coupled with sufficient data for the model to learn from. Segmentation at its core is a classification problem at the level of the voxel, which is the smallest unit of an image. The voxel is determined by the image section thickness and the spatial resolution at which the image is acquired, and lesions or whole organs consist of thousands of voxels. The density of data in segmentation is much higher compared to that in traditional classification tasks such as radiomics and thus is a job well suited for ML models [37].

In a study by Leung et al., a self-configuring DL framework was developed to perform fully automated, whole-body tumor segmentation and prognosis on PET/CT. These scans included patients with a wide variety of cancers: lung cancer, melanoma, lymphoma, head and neck cancer, breast cancer, and prostate-specific membrane antigen (PSMA) PET/CT scans of patients with prostate cancer. This deep, semi-supervised transfer learning approach yielded true-positive rates of 0.75, 0.85, 0.87, and 0.75 for patients with lung cancer, melanoma, lymphoma, and prostate cancer, respectively, on the tumor segmentation task. The risk score for head and neck cancer was significantly associated with overall survival by Cox regression analyses (*p* < 0.05) [38]. Coupled with predictive models for breast cancer, it accurately predicts the treatment response, showcasing the ability of a singular DL algorithm to determine diagnosis and prognosis across six different cancer types. This study was conducted using high-score PSMA lesion scans, but further evaluation of lower-score PSMA scans may provide a further insight into the true positivity the current models found to be indeterminate.

The MedSAM foundation model’s transformer-based architecture uses bounding-box prompts to guide segmentation, reducing annotation time by 82% compared to manual methods while maintaining robustness across diverse cancer types and imaging modalities [39]. It was novel in utilizing a foundation model, and instead of being confined to task-specific segmentation, it trained on 1.57 million medical images across 10 imaging modalities and over 30 cancer types. It achieved a median DSC of 0.91 across 86 internal validation tasks and 60 external validation tasks, showcasing better accuracy than modality-wise specialist models. However, performance varies for underrepresented modalities (e.g., mammography) and branching structures like vessels, highlighting the need for targeted fine tuning. In contrast, the VB-Net model specialized in brain metastasis segmentation, leveraging a compressed 3D convolutional neural network trained on 10,338 lesions to achieve a DSC of 0.91 in a prospective multi-center trial [40]. When integrated into clinical workflows, VB-Net improved radiologists’ segmentation accuracy (DSC increase: 0.87 to 0.92) and reduced contouring time by 42%, with junior radiologists benefiting the most. The model’s real-time processing (<2 s per lesion) and consistent boundary delineation (average surface distance: 0.40 mm) make it particularly valuable for stereotactic radiosurgery planning. However, its reliance on gradient-echo MRI sequences and challenges with irregular or meningeal-adherent lesions suggest opportunities for improvement through multi-sequence training. Both studies illustrate AI’s role as an augmentative tool rather than a replacement, with MedSAM enabling broad applicability across cancer types and VB-Net optimizing precision for a high-impact clinical task. Key limitations include modality-specific performance gaps and the need for human oversight in complex cases. Future directions should focus on expanding validation across diverse imaging protocols, integrating AI into picture archiving and communication systems (PACSs), and addressing rare anatomical variants through advanced architectures like vision transformers [39,40]. These advancements promise to enhance diagnostic workflows, reduce inter-observer variability, and support personalized treatment planning in oncology.

## 4. Treatment

Accurate prediction of disease progression and response to treatment in oncology is crucial. Early identification of therapy and the consequent administration of therapeutics can have a great impact on clinical outcomes. AI, along with DL strategies, may be a promising field in tumor response prediction to various treatments. Through the integration of imaging with clinical data, AI may enhance the administration of personalized treatment [27].

In hepatocellular carcinoma (HCC), Lin et al. developed a deep learning model that analyzes pre-treatment multiphase CT scans to forecast tumor response to transarterial chemoembolization (TACE) [41]. This model achieved an AUC of 0.91 and 80% accuracy in external validation, substantially outperforming traditional radiomics and BCLC staging systems by relying only on standard arterial-phase imaging, making it readily deployable in clinical workflows. Their model, however, is limited by a small sample size, low-resolution imaging, the exclusion of clinical data, and a lack of feature interpretability, all of which may affect robustness and clinical applicability. Further exploration to address these limitations can improve clinical efficacy by identifying likely non-responders, avoiding ineffective interventions, and promoting more efficient, targeted use of locoregional therapy.

Kuhn et al. introduced machine learning models to assist in preprocedural selection for portal vein embolization (PVE) in patients with colorectal cancer liver metastases (CRLM) [42]. Their models predicted both future liver remnant (FLR%) and kinetic growth rate (KGR%), advancing beyond traditional anatomical metrics to incorporate a functional treatment response. With these models, external AUCs of 0.88 and 0.69 were achieved, respectively, enabling patient-specific preoperative planning by integrating radiomics, statistical shape modeling, and laboratory data. Additionally, these machine learning models outperformed conventional multivariate models and achieved generalizability across institutions, advancing the precision of surgical risk assessment. This study is limited by the use of fixed cutoff predictions rather than continuous outputs, omission of key biological factors like KRAS mutation status, exclusion of patients not selected for PVE, and potential bias introduced by precomputed statistical shape model eigenvalues, all of which may affect the generalizability and clinical flexibility of the model. Future studies should aim to investigate the limitations, as these tools can help clinicians identify which patients are likely to benefit from PVE and curative resection, reducing unnecessary procedures and improving surgical planning.

In prostate cancer, Saha et al. demonstrated that AI-enhanced MRI interpretation can support biopsy triage by improving the detection of clinically significant disease while reducing false positives [43]. Using data from over 10,000 mp MRI scans across 45 centers, they found that their model outperformed 62 radiologists (AUROC 0.91 vs. 0.86), detected 6.8% more clinically significant prostate cancers, and reduced false positives by 50.4%. The retrospective and mixed-source dataset, use of a controlled online reading environment that may not reflect clinical practice, verification bias due to varied diagnostic standards, lack of diversity data such as patient ethnicity, and limited generalizability given that over 93% of MRIs were acquired from a single manufacturer are all avenues which warrant further investigation to increase the external validity of these results. Despite these limitations, these capabilities not only reduce unnecessary biopsies and overtreatment but also help guide treatment decisions between curative intervention and active surveillance, especially in resource-limited or low-volume centers.

Oshino et al. developed a machine learning model that predicts axillary lymph node metastasis in early-stage breast cancer using contrast-enhanced and B-mode ultrasonography [44]. The model’s high accuracy (AUC 0.93) offers a non-invasive alternative to sentinel lymph node biopsy, allowing for de-escalation of surgical intervention in appropriately selected patients and reducing morbidity. However, this study’s retrospective, single-center design without external validation; potential overfitting due to a small dataset; and reliance on physician-determined features rather than an end-to-end model all limit the generalizability of these results.

Radiomics is another field which can improve treatment outcomes with high-level feature extraction from images and prediction of treatment response in oncology patients. Radiomic models predict this by analyzing tumor and spatial heterogeneity [45]. Radiomic models have also predicted responses to neoadjuvant chemotherapy and chemoradiotherapy accurately in several cancers. Radiogenomics further integrates imaging and genomic data, enhancing prognostic accuracy. AI-powered radiogenomic models can predict tumor mutations, treatment resistance, and molecular subtypes, facilitating more personalized therapeutic strategies [46]. This integration is particularly relevant in precision oncology, where AI can optimize patient selection for targeted therapies based on an individual’s molecular and imaging profile.

There are various studies that incorporate radiomics model prediction and treatment response, such as the ultrasound-based deep learning radiomics nomogram (DLR_Nomogram) used to assess malignant risk in ovarian tumors. A radiomics nomogram is a visual predictive tool that combines quantitative radiomic features to estimate treatment response. One study validated the utility of DLR_Nomogram against the ovarian-adnexal reporting and data system (O-RADS). In assessing general ovarian tumors, it achieved AUC values of 0.985 in training and 0.928 in testing. For tumors classified as O-RADS 4 and 5, the DLR_Nomogram achieved AUC values of 0.955 in training and 0.869 in testing. The AUC value of its testing set was lower compared to that of O-RADS, but the difference was not significantly significant [47]. In another study, deep learning was utilized to integrate MRI images to predict bone metastases in primary prostate cancer. The best prediction model that utilized radiomics had an AUC of 0.93, demonstrating good net clinical benefit and its ability to serve as a valuable predictor of risk [48]. MRI radiomics has also predicted a complete pathological response in rectal cancer patients who were being treated with chemoradiotherapy. A meta-analysis conducted by Kao and Hsu showed studies applying radiomics for the prediction of treatment response in esophageal cancer had a pooled AUC of 0.813 [49]. It demonstrated the strong predictive capabilities of radiomics models. Additionally, multimodal approaches using PET, CT, and MRI have shown increased predictive power. One study which involved patients with esophageal cancer treated with chemoradiation therapy demonstrated that integrating CT-based radiomics and dosimetric parameters achieved higher accuracies from 0.625 to 0.708 and AUCs from 0.412 to 0.689, compared to those of radiomics alone [45]. Delli Pizzi et al. showed that in locally advanced rectal cancer, MRI radiomics combined with ML enhanced the prediction of treatment response, thus demonstrating the multimodal possibility of AI-based systems in cancer [50].

These advancements reflect a significant shift in the role of AI from diagnostic augmentation to therapeutic navigation. By transforming routine imaging into predictive, patient-specific decision tools, AI is actively influencing how and for whom interventional procedures are performed.

## 5. Technological Advancements

### 5.1. Imaging Optimization

AI has been important in increasing accuracy and precision in interventional oncologic imaging. High-quality imaging is critical for cancer diagnosis and guiding procedures such as tumor ablation, trans-arterial chemoembolization, and targeted biopsy. However, traditional imaging is often degraded by artifacts, noise, and low resolution, which hinder diagnosis and procedural efficiency. AI has revolutionized image optimization, enhancing lesion visibility and enabling real-time decision-making [51]. AI-driven low-dose imaging protocols can reduce radiation exposure by 36–70% without compromising diagnostic quality, with some studies achieving up to 95% dose reduction in pediatric radiology [52]. In CT, AI algorithms optimize patient positioning and scan range selection, reducing radiation exposure by up to 21% by avoiding overscanning [53].

Despite these advancements, AI applications in radiology face challenges, including limited generalizability due to algorithms’ dependence on specific scanner models and acquisition parameters [53]. Many AI models are trained on narrow datasets, raising concerns about their reliability when applied to diverse patient populations or imaging protocols [53]. Additionally, the “black-box” nature of deep learning models often results in insufficient explainability, undermining clinical trust and adoption [53].

AI-augmented techniques have significantly improved imaging quality in interventional oncology by reducing noise and removing artifacts. Imaging heterogeneities and motion artifacts degrade MRI and CT quality, complicating tumor characterization. DL has proven to be highly effective at noise reduction and artifact removal, improving image readability. For instance, AI-based noise reduction increases the signal-to-noise ratio (SNR) in MRI, enhancing hepatic cancer detection by 7.3% [51]. Hybrid AI systems combining post-processing and real-time acquisition optimization maximize image quality while minimizing radiation doses [52]. In MRI, AI-based motion correction techniques, including GANs, successfully reduce artifacts from unintentional patient or fetal movement, achieving a structural similarity index of 93.7% and a peak signal-to-noise ratio of 33.5 dB [53]. However, retrospective motion correction methods, while efficient, may not fully restore image integrity if motion corruption is severe, and they depend heavily on the training data quality. Furthermore, AI solutions for artifact removal often lack external validation, limiting their real-world applicability [53].

Super-resolution AI is another transformative advancement, using DL to convert low-resolution scans into high-definition images with enhanced tumor and anatomical detail. This technique improves liver metastasis detection, enabling better lesion segmentation and volumetry, with AI-enhanced images scoring 4.317 ± 0.72 compared to 3.223 ± 0.985 for conventional T2 FS (*p* < 0.0001) [54]. AI-driven super-resolution GANs (SRGANs) combined with transformer-based architectures achieve up to fourfold resolution enhancement in MRI and CT without introducing noise [52].

Contrast optimization is another area where AI excels. AI-driven algorithms adjust the pixel intensity distribution in real time, improving lesion visibility in contrast-enhanced MRI and CT scans. These advancements are particularly valuable in hepatocellular carcinoma (HCC) imaging, where AI-enhanced contrast differentiation improves diagnostic accuracy. Reinforcement learning dynamically optimizes contrast levels, boosting lesion detectability in PET scans by 15–20% compared to that in standard methods [52]. In MRI, AI reduces gadolinium-based contrast agent (GBCA) doses by 80–90% while maintaining diagnostic quality, addressing safety concerns for patients with renal impairment [53]. Nevertheless, AI-based contrast reduction techniques may struggle with heterogeneous tumor enhancement patterns, potentially leading to false negatives in poorly vascularized lesions. Additionally, while AI can minimize contrast doses, the long-term clinical impact of synthetic contrast-enhanced images requires further validation.

DL has also accelerated imaging acquisition without sacrificing diagnostic accuracy. AI-enhanced T2-weighted MRI sequences produce sharper images with more prominent lesions, reducing scan times by 71% (from 178.9 ± 85.3 s to 51.23 ± 10.1 s) [35]. AI-based reconstruction of undersampled MRI data, such as recurrent CNNs, achieves high-quality images even with elevenfold undersampling, significantly speeding up acquisition [53]. However, ultra-fast MRI reconstructions may introduce subtle artifacts or blurring in complex anatomical regions, necessitating radiologist oversight. Moreover, vendor-specific AI solutions lack interoperability, restricting their use across different imaging platforms.

Automated segmentation algorithms based on DL standardize lesion measurement, reducing interobserver variability. These tools enhance liver metastasis detection and quantification, improving treatment monitoring. AI models achieve Dice Similarity Coefficient (DSC) values ≥ 0.85 in segmentation tasks, ensuring precise anatomical delineation in CT and MRI [52]. Despite their precision, segmentation algorithms may fail in cases of atypical tumor morphology or poorly defined boundaries, requiring manual correction [53]. Additionally, the integration of AI into clinical workflows remains challenging due to regulatory hurdles and the need for continuous model updates.

### 5.2. Automated Reporting

Medical reporting remains a fundamental yet increasingly burdensome aspect of clinical practice, exacerbated by the exponential growth of electronic health records (EHRs) and the cognitive load they impose, with nearly 75% of physicians citing EHRs as a significant contributor to burnout [55]. Discrepancies between documented reports and actual imaging findings further compound these challenges, with studies revealing clinically significant errors in 27.2% of operative procedural reports [56]. The advent of AI-driven automated reporting offers a transformative solution to these inefficiencies. Natural Language Processing (NLP) and ML can parse complex clinical data, extract relevant findings, and generate structured reports with high accuracy, significantly reducing administrative burdens and minimizing documentation errors [57]. For instance, recent advancements like Flamingo-CXR, a vision-language model fine-tuned for chest X-rays, demonstrate a 33% improvement in clinical accuracy (CheXpert F_1_ score) over prior state-of-the-art systems, with 77.7% of AI-generated outpatient reports rated by radiologists as equivalent or preferable to human-generated reports [58]. This capability is particularly critical in addressing the “satisfaction of report errors”, where prior inaccuracies are perpetuated in subsequent documentation, as highlighted by Pesapane et al. [59]. AI models like Flamingo-CXR standardize language while retaining clinical nuance (e.g., disease severity and anatomical location), reducing variability and improving overall report quality. Notably, in cases with no abnormalities, AI-generated reports achieved a 94% equivalence/preference rate, allowing radiologists to prioritize complex cases [58].

Recent work in advancing technology underscores how AI-powered NLP tools are now being integrated into radiology workflows to pre-populate reports with AI-generated findings, expediting the reporting process and improving consistency [60]. These systems can automatically flag critical findings (e.g., pneumothorax or fractures) and prioritize them for radiologist review, enhancing efficiency in high-volume departments. For example, AI-driven “automated hanging protocols” pull prior imaging and EHR data into the reporting interface, reducing time spent on manual searches and providing contextual insights at the point of diagnosis [60]. Additionally, AI assists in structured reporting, organizing unstructured dictations into standardized formats, which minimizes variability in follow-up recommendations and improves overall report quality.

Recent developments highlight the use of large language models (LLMs) like RadGPT which segment tumors and generate detailed structured and narrative reports, including tumor size, shape, and interactions with adjacent structures. Such tools not only streamline workflows but also enhance diagnostic precision by transforming imaging data into comprehensive reports. However, AI systems like GPT-4 still lag behind human radiologists in generating coherent and comprehensive “Impressions” sections, indicating areas for improvement.

However, AI is not infallible. A striking finding from recent research revealed that 24.8% of cases contained clinically significant errors in both AI- and human-generated reports, though the error types often differed. AI struggled with spatial reasoning (e.g., mislocating lung opacities), while humans overlooked subtle findings or contextual details [58]. This divergence underscores the potential for synergy in clinician–AI collaboration. When radiologists edited AI-generated drafts, the revised reports were preferred over human-only reports in 53.6% of intensive care cases, demonstrating the utility of AI as an assistive tool rather than a standalone solution [58]. Recent work highlights that such collaboration is most effective when AI systems are seamlessly integrated into existing radiology workflows, such as PACS and EHRs, though interoperability challenges persist across vendors and institutions [60].

Challenges remain, such as the need for multimodal integration (e.g., incorporating lateral X-ray views, prior scans, and EHR data to improve accuracy) and regional adaptation to local reporting styles (e.g., structured templates in India vs. free-form narratives in the U.S.) [58]. Recent work also emphasizes the importance of explainable AI (XAI) to address the “black box” problem, ensuring that radiologists understand how AI arrives at conclusions to build trust and facilitate clinical adoption [60]. Advancements may leverage radiomics. AI-driven analysis of quantitative imaging features to predict disease progression or treatment responses, further personalizing reporting. The future of automated reporting likely lies in hybrid workflows, where AI drafts are refined by radiologists, balancing efficiency with expert oversight. As AI capabilities evolve, their role in mitigating burnout, reducing errors, and enhancing report quality will expand [58].

### 5.3. Complex Data Integration

With an ever-increasing number of imaging data points, number of new complex therapies, and volume of encounters, the importance of consolidating this information is paramount [61]. Many of these data points exist in isolation, however, meaning that one diagnostic technology, such as a CT scan, may not directly interact with another. AI integration can allow for the rapid and efficient corroboration of multiple data points to provide a more cohesive and focused differential for patients undergoing cancer management. Automated AI systems can cross-reference imaging reports within the EHR with laboratory values, genetic profiles, and prior treatment histories to generate more comprehensive diagnostic assessments [62]. [N] AI-powered image fusion techniques, such as those combining CT, MRI, and ultrasound, enable clinicians to synthesize multimodal data into a unified diagnostic framework, significantly enhancing precision in complex cases like tumor detection and vascular anomalies [63].

In cardiac imaging, AI tools are being developed to integrate multimodal data (e.g., EHRs, imaging protocols, and genomic data) to optimize test selection, protocoling, and risk stratification, though most remain at early Technology Readiness Levels (TRLs 1–3) [64]. Additionally, AI-powered decision-support tools can assist multidisciplinary tumor boards by synthesizing relevant patient data and highlighting key prognostic factors in real time [65]. For instance, AI-driven predictive models in interventional oncology leverage radiomics and deep learning to automate tumor segmentation, simulate treatments, and predict outcomes, though clinical adoption remains limited due to validation gaps [66].

AI-driven orchestration platforms are emerging to automate sequential algorithm execution (e.g., plaque quantification followed by risk prediction) and integrate outputs into PACS/EHRs, though interoperability challenges persist [64]. However, a 2024 meta-analysis found that while 67% of studies reported reduced task times with AI, real-world workflow integration often fails to achieve significant efficiency gains due to heterogeneity in implementation (e.g., concurrent vs. sequential reading workflows) [64]. The integration of AI with robotic systems in interventional radiology (IR) exemplifies this challenge, where real-time data fusion (e.g., needle tracking and image overlay) improves procedural accuracy but requires seamless interoperability with existing imaging hardware [67].

A study by Ye et al. illustrated the use of multimodal data integration and DL by developing a non-invasive image biomarker for predicting the response of oncological treatments on non-small-cell lung carcinoma (NSCLC) [68]. By using AI features to integrate several different contrast and non-contrast CT data points, they were able to construct a fused model and a novel imaging-based biomarker. This further illustrates how integrating multiple data streams allows for a more precise and personalized approach to cancer diagnosis and treatment. Similarly, AI-enhanced synthetic contrast imaging (e.g., generating contrast-like CT scans from non-contrast inputs) reduces reliance on invasive agents while maintaining diagnostic quality, though validation for high-stakes procedures like renal cancer interventions is ongoing [69].

In cardiac CT, AI algorithms combine plaque quantification, CT-derived fractional flow reserve, and clinical data to improve prognostic accuracy (TRLs 5–9), though generalizability remains limited by single-center datasets [64]. Notably, AI tools used as “second readers” in detection tasks (e.g., pulmonary embolism on CT) often extend interpretation times when radiologists must reconcile AI outputs with their own assessments, highlighting the need for seamless workflow integration [64]. The EU AI Act’s emphasis on transparency and human oversight (e.g., ESR guidelines) underscores the importance of balancing automation with clinician control, particularly in high-risk IR applications like autonomous catheter navigation [70].

Radiological data and pathological reports are often integrated together to provide a more comprehensive clinical picture in the treatment of cancer. By linking radiomic and pathomic data, AI can identify tumor subtypes, predict treatment response, and refine prognostic models. A study by Wang et al. demonstrated the ability of a combined nomogram ML model in integrating pathomics, radiomics, and immunoscore data in predicting the overall survival (AUC = 0.86) and disease-free survival (AUC = 0.875) in patients with colorectal lung metastasis [71]. AI’s role in interventional oncology extends to real-time intraprocedural guidance, where it merges imaging with genomic data (e.g., radiogenomics) to personalize ablation strategies, though ethical concerns about data privacy persist [72].

In cardiac MRI, AI-enabled fusion of pixel-based imaging data with clinical variables (e.g., EHR-derived risk factors) has shown promise for predicting major adverse cardiac events, but real-world deployment is hindered by retrospective study designs and the lack of longitudinal monitoring [64]. Studies have also shown that AI-assisted analysis of both imaging and pathology data can improve accuracy in predicting which patients will respond to immunotherapy, a treatment that currently lacks reliable biomarkers in many cases [73]. However, AI’s reliance on large, labeled datasets remains a barrier in IR, prompting exploration of synthetic data (e.g., CT-derived fluoroscopy simulations), which require rigorous validation to avoid biases [74].

However, Mastrodicasa et al. cautioned that AI-reconstructed images may introduce “hallucinations” (fabricated features) or omit critical details (“reverse hallucinations”), necessitating rigorous validation before integration into clinical workflows [64]. This aligns with challenges in IR, where AI-driven robotic systems (e.g., for CT-guided biopsies) must undergo extensive calibration to ensure safety and reliability across diverse patient anatomies [75].

The persistent issue of data silos remains a critical barrier, as most AI tools are trained on single-center datasets, limiting generalizability. Large, diverse datasets (e.g., UK Biobank) are needed to reduce bias and improve interoperability [76]. The slow adoption of AI in IR compared to diagnostic radiology further highlights specialty-specific hurdles, such as the need for real-time, high-stakes validation [77]. Workflow integration challenges also persist; while AI can streamline cardiac MRI workflows (e.g., automated biventricular segmentation, TRLs 8–9), deployment requires alignment with hospital IT infrastructure and clinician oversight [76]. In IR, AI’s potential to automate collimation and reduce radiation exposure is counterbalanced by integration challenges in fast-paced environments [78]. Finally, ethical and legal barriers, including regulatory fragmentation (e.g., the FDA vs. the EU AI Act) and the environmental costs of AI training (e.g., energy-intensive data centers), complicate widespread adoption [76].The ESR’s call for standardized AI literacy and post-market monitoring in radiology reflects the urgent need for global harmonization in IR-specific AI governance [70].

## 6. Limitations

### 6.1. Clinical Limitations

**Figure 3:**
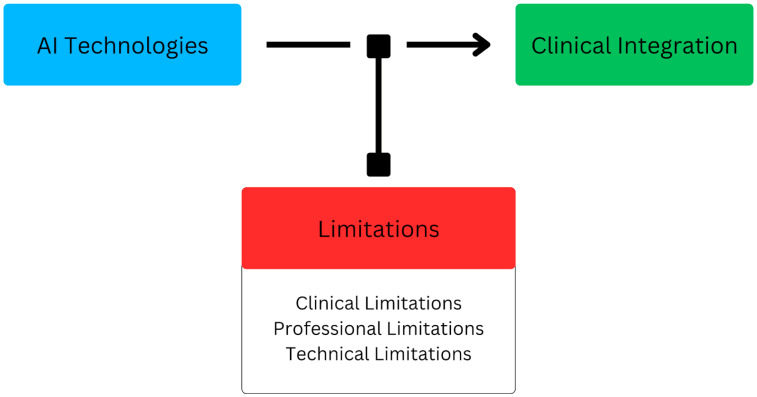
Summary of AI limitations in cancer imaging applications.

This review highlights the potential applications of AI in cancer imaging, and as such, AI and ML tools need to address a specific clinical challenge (Figure 3). AI developers should understand and be cognizant of the clinical setting that these tools would be utilized in. In order to bridge that gap, a practicing clinician should be intimately involved with the development process of AI tools.

By applying sophisticated ML algorithms and computational intelligence, AI can revolutionize cancer image analysis. Currently, diagnostic and analytical processes have centered around organization-specific pathways and protocols, potentially leading to bottlenecks in analysis simply due to the sheer amount of information produced by “omics” technologies. AI methods can enable a shift from organization-centric to patient-centric healthcare, which may improve clinical outcomes and reduce healthcare costs by revealing better individualized solutions [79].

Computerized oncological image analysis is enabling the transition from qualitative image analysis to quantitative assessment using automated methods with the goal of earlier detection, enhanced lesion characterization, and the provision of more effective decision support tools [80]. While some current AI and ML tools have the ability to do so, there are still hurdles for these tools to overcome, such as the need for reproducible and reliable tumor segmentation, accurate computer-assisted diagnosis, and clinically useful prognostic biomarkers. A specific challenge is the tracking and quantification of intra-/inter-tumoral heterogeneity throughout the course of the disease [81]. In order to achieve this goal, access to large-scale longitudinal imaging datasets is required.

**Figure 4:**
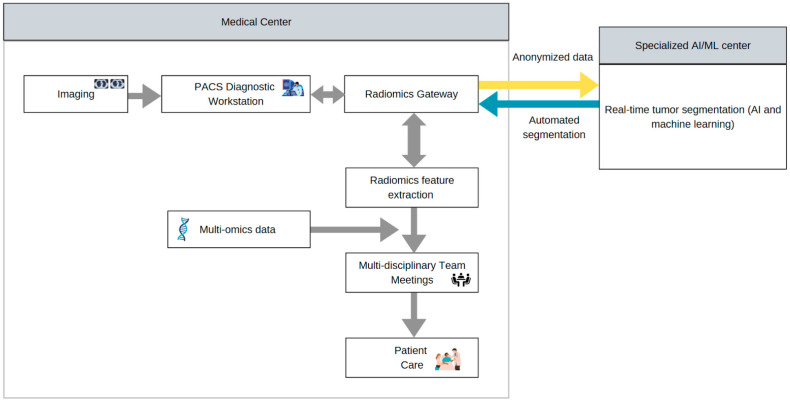
Real-time tracking workflow of tumor segmentation with multi-omics data integration for precision oncology.

A field of medicine that AI and ML can be particularly revolutionary for is precision oncology: the determination of a patient’s therapy derived from their tumor’s molecular profile (Figure 4). The ideal solution involves large-scale data collection from multiple institutions that can also anticipate privacy issues and emphasize patient confidentiality, while also supporting longitudinal learning [82]. The foremost obstacle is bridging the gap between nascent AI tools and clinical practice, which can be achieved with well-validated clinical research studies of such AI applications. Only then could these AI tools be deployed in precision oncology, with the end goal of decreasing the cost of precision oncological treatments through more accurate patient selection processes [83].

### 6.2. Professional Limitations

In addition to the challenges within the clinical domain, challenges in the professional world dictate the real-world deployment of ML in cancer imaging. The increase in demand for imaging compounded with acute and chronic workforce shortages can lead to greater radiologist stress and burnout. IT departments will need to optimize infrastructure for a greater workload with more complex nuances. The radiological workforce itself will need to accept the role of AI and ML in the clinic, considering both the threats and opportunities associated with the use of such technologies [5].

An online survey of 569 radiologists from 35 countries was conducted in preparation for an AI and ML in Cancer Imaging meeting organized by the International Cancer Imaging Society and the Champalimaud Foundation in 2019. It showed that the majority of radiologists perceived the benefits of AI to outweigh the potential risks. Most agreed that the pros of AI included alerting radiologists to abnormal findings, increasing work efficiency, making diagnostic suggestions in the event of uncertainty, accepting that the radiologist should be responsible when a mistake is made, and increasing direct communications with patients. The respondents felt that it was unlikely that AI and ML technologies will replace the job of a radiologist. The same majority found it necessary to continue to prepare for the arrival of AI by investing in education, testing new techniques, working with commercial vendors in AI development, and supporting the curation of image data at scale [5].

Additionally, the real-world implementation of AI in the clinical workspace will need to be addressed. Radiographers and technicians will require training for a better understanding of AI. Healthcare systems will need to account for the regulatory approval processes, as they can be complicated, expensive, and nebulous. Finally, an informatics team will need to develop the platform on which the AI tools can be developed and tested on and create the required imaging and data repositories [5].

### 6.3. Technical Limitations

In addition to clinical and professional challenges, there are several technical challenges that must be addressed to further advance deep learning and multi-omics integration. One such limitation includes processes which require time-consuming expert annotations from radiologists and pathologists for 3D segmentation. This significantly limits the availability of large, high-quality datasets for training deep learning models [84]. Label noise in oncological imaging data (e.g., inter-observer variability) in tumor annotations introduces another uncertainty that complicates model development [84]. Furthermore, an important limitation is the lack of automatic integration of radiomic and genomic data. Even though databases like The Cancer Genome Atlas (TCGA) and The Cancer Imaging Archive (TCIA) provide valuable imaging and genomic data, their information must be processed and integrated manually by non-standardized procedures, which decreases the efficiency of correlating the genotype with the phenotype [53]. This is particularly important in oncological imaging, where the correlation between specific genes and the phenotype appearing is crucial, and it further helps in personalized treatment, increasing efficiency [85]. Additionally, computational constraints hinder the analysis of the entire image, particularly in high-resolution oncologic imaging (e.g., whole-slide pathology scans), where memory limitations bar the use of spatial context within deep learning algorithms [84]. These challenges will be overcome through standardized annotation protocols, noise-robust algorithms, seamless multi-omics integration platforms, and computing infrastructure innovation to leverage the full potential of AI-assisted oncology research [84,85].

## 7. Future Directions

The rapid evolution of AI holds a promising future for its future integration into the clinical workflow as the technology continues to integrate itself to work in conjunction with radiologists. AI will likely continue the trend of micro-optimizations focused on greater efficiency, as its narrow clinical usage means each system excels at one specific task. As these systems continue to expand, existing optimizations can be combined to enhance cancer screening [86].

Initially, AI techniques which augment non-diagnostic tasks will demonstrate utility in the clinical setting [2]. These tasks include radiology reporting, in which tools like ChatGPT-3.5 and 4 and assistive AI have significant potential to enhance accuracy and standardization by generating impressions from radiology findings [87]. Within disease detection, the integration of advanced AI techniques such as reinforcement learning, graph neural networks, and self-supervised learning could enhance model performance [88].

Another area of evolution lies in improvements in personalized medicine, where AI can be used to tailor patient-specific treatment strategies through patient stratification, treatment optimization, and customized prediction for better patient outcomes [88]. An emphasis on patient accessibility must be placed to ensure strong doctor–patient communication throughout the cancer treatment timeline. Diagnostic imaging reports are generally written with medical jargon and technical details for accurate communication between medical providers. AI has the potential to simplify imaging reports without significant disruptions for patients to gain a better understanding of their cancer diagnosis [89]. While further research must be conducted on its application in radiology, AI’s efficacy in simplifying patient reports has recently been demonstrated in pathology reporting, providing evidence of improved understanding and reduced communication time [90]. The implementation of these technologies highlights the potential of AI to create a more patient-centric approach to cancer treatment by bridging the gap between medical providers and patients.

Resource-poor health institutions face limitations in equipment availability, personnel expertise, and infrastructure. AI can further aid patients in healthcare accessibility by delivering more equitable cancer care through more inclusive datasets, integrating social determinants of health, and developing ethical frameworks [91]. Investments in clinical radiology education, infrastructure, and AI systems can help promote more positive patient outcomes [92].

## 8. Conclusions

AI is revolutionizing oncological imaging in every stage of the cancer treatment timeline by enhancing screening, diagnosis, and treatment response. As imaging demand increases, AI-driven tools help radiologists streamline workflows, reduce errors, and improve efficiency. Automated reporting and complex data integration are reshaping how imaging data is utilized, leading to earlier cancer detection and more personalized treatment strategies.

Despite these advancements, challenges remain in the widespread adoption of AI in clinical practice. Issues such as poor data standardization, lack of generalizability, and patient privacy concerns must be addressed to ensure reliability and clinician trust. Regulatory oversight and prospective validation studies will be crucial in refining AI applications and ensuring their safe integration into healthcare systems.

Moving forward, AI’s role in imaging will continue to expand, complementing radiologists rather than replacing them. By refining diagnostic accuracy, optimizing treatment planning, and improving patient outcomes, AI has the potential to redefine the landscape of cancer care, making it more precise, efficient, and patient-centered.

## Data Availability

All images included in the review are the property of the authors.

## Abbreviations

The following abbreviations are used in this manuscript:

AI	Artificial Intelligence
ML	Machine Learning
DL	Deep Learning
CT	Computed Tomography
PET	Positron Emission Tomography
AUC	Area Under the Curve
CLAIM	Checklist for Artificial Intelligence in Medical Imaging
TNBC	Triple-Negative Breast Cancer
PMSA	Prostate-Specific Membrane Antigen
PACS	Picture Archiving Communication System
SNR	Signal-to-Noise Ratio
HCC	Hepatocellular Carcinoma
TACE	Transarterial Chemoembolization
PVE	Portal Vein Embolization
CRLM	Colorectal Liver Metastasis
EHR	Electronic Health Record
NLP	Natural Language Processing
LLM	Large Language Models
NSCLC	Non-Small-Cell Lung Carcinoma
TCGA	The Cancer Genome Atlas
TCIA	The Cancer Imaging Archive
